# Successful surgical management of a combined abdominal and thoracic penetrating injury: a case report

**DOI:** 10.1093/jscr/rjad245

**Published:** 2023-05-13

**Authors:** Zamaan Hooda, Lisa O’kane, John Paul Bustamante, Bledi Zaku, Jideofor Aniukwu, Scott Wessner, Luis Cerda

**Affiliations:** Department of Surgery, St. Joseph’s University Medical Center, Paterson, NJ, USA; Department of Surgery, St. Joseph’s University Medical Center, Paterson, NJ, USA; Department of Surgery, St. Joseph’s University Medical Center, Paterson, NJ, USA; Department of Surgery, St. Joseph’s University Medical Center, Paterson, NJ, USA; Department of Surgery, St. Joseph’s University Medical Center, Paterson, NJ, USA; Department of Surgery, St. Joseph’s University Medical Center, Paterson, NJ, USA; Department of Surgery, St. Joseph’s University Medical Center, Paterson, NJ, USA

## Abstract

Penetrating rebar injuries are extremely rare occurrences, but they are very life-threatening, particularly when involving the thoracic and abdominal cavities. The surgical approach to these traumatic injuries depends upon the length and diameter of the rebar as well as the path of penetration into the abdominal and thoracic regions. Due to the highly uncommon occurrence of penetrating rebar injuries, there is very limited information and studies pertaining to this topic in the literature. In this case report, we present a 43-year-old male patient sustaining a rebar penetrating injury, with the entry site being the left flank and the exit site being the anterior left chest. Upon arrival, the patient was emergently taken to the operating room and underwent simultaneous exploratory laparotomy and a left thoracotomy. The operation was successful in removing the rebar and the patient survived.

## INTRODUCTION

Trauma to the abdomen is considered to be penetrating when an object violates the peritoneal cavity, and established guidelines describe operative management in such events [1]. Similarly, approaches to the operative interventions have been greatly described in the literature regarding penetrating thoracic trauma [2]. However, there are no established standards and strategies for combined abdominal and thoracic penetrating injuries, particularly those that involve impalement with a steel bar or rebar [3, 4]. The lack of recommended approaches is secondary to the extremely rare occurrence of rebar thoracoabdominal penetrating injuries, with only a few reports describing this clinical phenomenon [3–6].

When such an injury is encountered, the method of intervention poses a unique challenge to surgeons. The surgical approach depends heavily on the length and diameter of the rebar as well as on the path into the patient [4, 7, 8]. This case report describes the successful surgical management of a thoracoabdominal rebar penetrating injury.

## CASE REPORT

**Figure 1:**
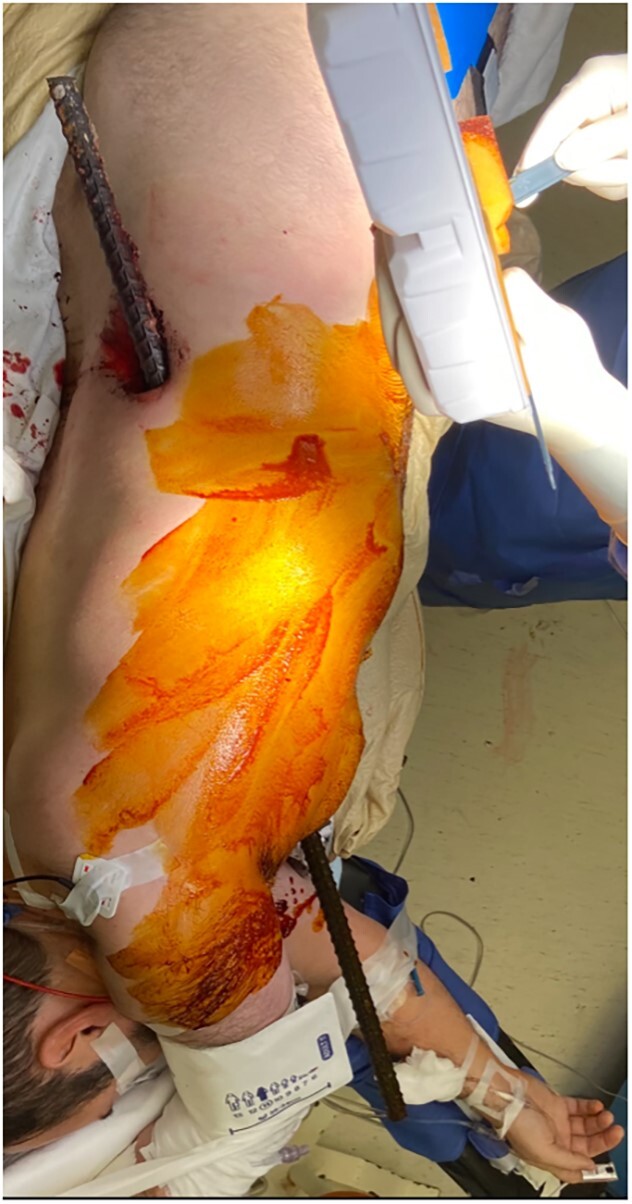
Rebar penetrating through the left flank through the left anterior chest.

**Figure 2:**
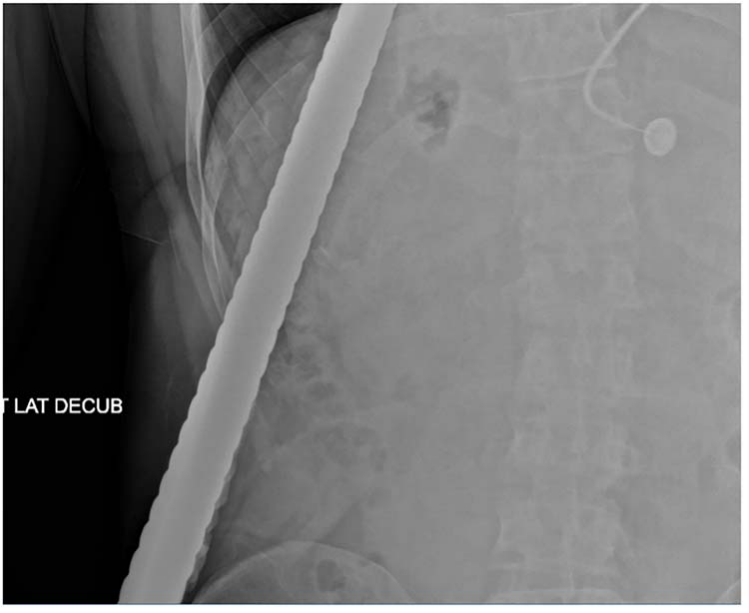
Abdominal X-ray demonstrating the rebar trajectory into the left anterior chest; the path is distorted secondary to patient positioning during imaging.

A 43-year-old male patient presented to the emergency department after falling onto an exposed rebar at work at a construction site. The rebar entered the patient’s left flank and exited via the anterior aspect of the left chest. His initial blood pressure was 137/96 and his heart rate was 114 beats per minute. Physical examination revealed a rebar measuring ~80 cm impaled through the patient’s left flank and left chest (Fig. 1). While in the trauma bay, an abdominal X-ray was obtained, demonstrating a right-sided pneumothorax, and the rebar’s path was extending into the left anterior thoracic cavity (Fig. 2). Afterward, the patient was taken to the operating room where a left thoracotomy and exploratory laparotomy were performed.

**Figure 3:**
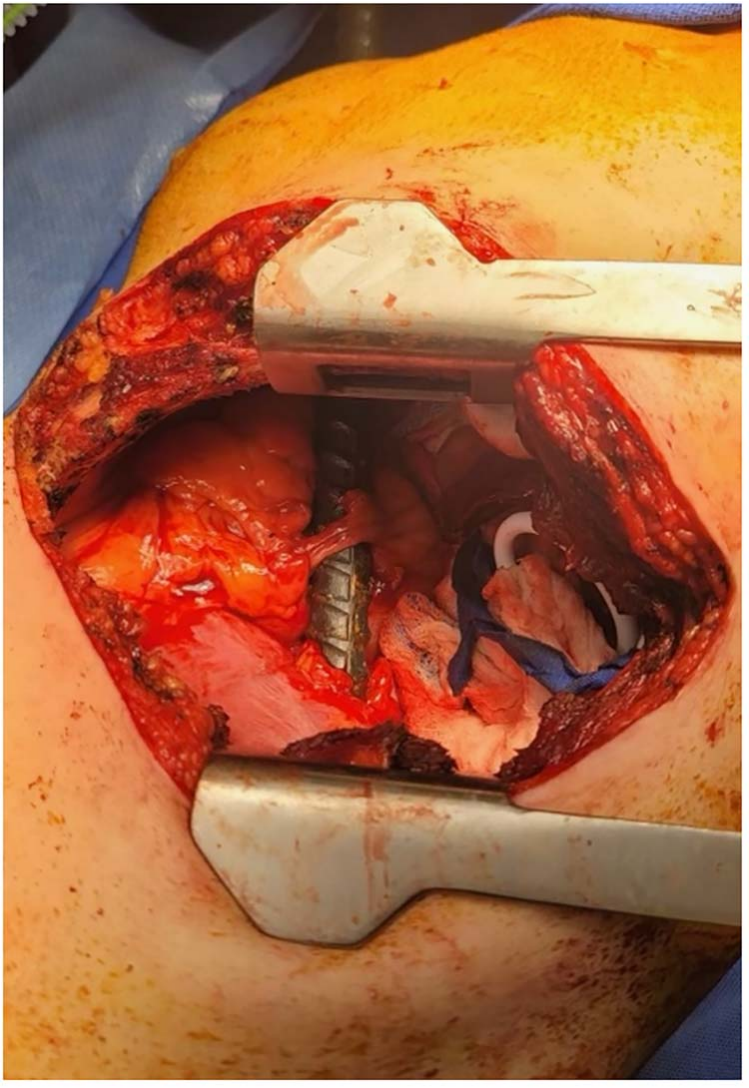
Thoracotomy incision revealing the rebar penetrating the diaphragm and coming in contact with the phrenic nerve.

Once in the operating room, the patient was placed in a semi-lateral, right-sided decubitus position exposing the left side of the chest. A left thoracotomy was performed in the sixth intercostal space and the pleural cavity was entered. At this time, we were able to visualize the rebar in the posteromedial left chest with a clear site of penetration through the diaphragm close to the pericardium and through the left anterior chest, and we were making contact with the phrenic nerve (Fig. 3). Intraoperative transesophageal echocardiogram confirmed no intrapericardial injury, and direct visualization revealed no pleural fluid. Afterward, a left subcostal incision was made in the abdomen, which demonstrated a posterolateral splenic injury. Digital control of the splenic hilum and thoracic bleeding were controlled and the rebar was removed through the right flank. Once removed, improved visualization revealed two separate diaphragmatic full-thickness lacerations, which were primarily repaired. Two chest tubes were placed and the thoracotomy incision was closed.

The patient was then placed in supine position for further exploration of the abdominal cavity. The spleen was found to have a laceration with moderate hemorrhage. The left upper quadrant was packed and the small bowel was inspected, revealing no injuries. There was no sign of gastric or colonic injuries. The packing was removed, and no further bleeding from the spleen was noted. An upper endoscopy was also performed, which did not demonstrate evidence of trauma. The incision was closed and the patient was transferred to the surgical intensive care unit.

Once stable, a computed tomography (CT) scan of the chest and abdomen was performed, further characterizing the splenic injury as Grade III with a 4.5-cm laceration with hematoma. This injury and L1–L3 spinous fractures also noted on CT imaging were nonoperatively managed. The following day, the patient was extubated, and after extensive rehabilitation and pain management, the patient was discharged 12 days after surgical intervention to a rehabilitation facility. The patient was seen in the surgical office 1 month after discharge and complained mostly of shortness of breath after walking long distances attributed to the deconditioning and loss of muscle mass after the traumatic incident. We expect the patient to completely recover and return to his previous state of health.

## DISCUSSION

Although rare, trauma involving impalement of the abdominal and thoracic cavities is extremely severe and is associated with high morbidity and mortality. Given the multiple potential life-threatening injuries that are associated with thoracoabdominal impalement injuries, surgeons are faced with a unique challenge with operative management. This task is made more difficult due to the lack of systemic guidance to follow when faced with such injuries, which is attributed to their rarity [3–6]. However, recommendations have been made by those able to successfully operate and manage patients in this clinical scenario. Outcomes of patients with impalement injuries can initially be enhanced by rapid transport of the patient to the nearest trauma facility. Upon arrival, it is imperative to assess the patient efficiently and perform a targeted examination, particularly with radiologic imaging, which should not delay definitive management. Throughout transport and examination, there should be an emphasis placed on avoidance of removing the impaled object, as this can cause further damage to organs and can lead to the loss of any tamponade effect [3, 9, 10].

The patient presented in this case report had a thoracoabdominal penetrating rebar injury that clearly had the potential to cause far more extensive damage than it did, had it taken a different trajectory into the patient. The successful outcome in this situation was also determined by the care provided to the patient, which reflected the above outlined techniques that we also endorse to other surgeons facing similar circumstances.
